# A Miniature Sucrose Gradient for Polysome Profiling

**DOI:** 10.21769/BioProtoc.4622

**Published:** 2023-03-20

**Authors:** Ansul Lokdarshi, Albrecht G. Von Arnim

**Affiliations:** 1 Department of Biology, Valdosta State University, Valdosta, GA 31698, USA; 2 Department of Biochemistry & Cellular and Molecular Biology, University of Tennessee, Knoxville, TN 37996-1939, USA

**Keywords:** Mini sucrose density gradient, Polysome profiling, Ultracentrifugation, Translation, Erb binding protein 1 (EBP1), Target of rapamycin (TOR), Ribosome fractionation, GCN2, Salt stress, *Arabidopsis* meristem

## Abstract

*Polysome profiling by sucrose density gradient centrifugation is commonly used to study the overall degree of translation (messenger RNA to protein synthesis). Traditionally, the method begins with synthesis of a 5*–10 mL sucrose gradient onto which 0.5–1 mL of cell extract is layered and centrifuged at high speed for 3–4 h in a floor-model ultracentrifuge. After centrifugation, the gradient solution is passed through an absorbance recorder to generate a polysome profile. Ten to twelve fractions (0.8–1 mL each) are collected for isolating different RNA and protein populations. The overall method is tedious and lengthy (6–9 h), requires access to a suitable ultracentrifuge rotor and centrifuge, and requires a substantial amount of tissue material, which can be a limiting factor. Moreover, there is often a dilemma over the quality of RNA and protein populations in the individual fractions due to the extended experiment times. To overcome these challenges, here we describe a miniature sucrose gradient for polysome profiling using Arabidopsis thaliana seedlings that takes ~1 h centrifugation time in a tabletop ultracentrifuge, reduced gradient synthesis time, and also less tissue material. The protocol described here can be easily adapted to a wide variety of organisms and polysome profiling of organelles, such as chloroplasts and mitochondria.

Key Features

• Mini sucrose gradient for polysome profiling that requires less than half the processing time vs. traditional methods.

• Reduced starting tissue material and sample volume for sucrose gradients.

• Feasibility of RNA and protein isolation from polysome fractions.

• Protocol can be easily modified to a wide variety of organisms (and even polysome profiling of organelles, such as chloroplast and mitochondria).

Graphical Overview

**Figure 1.**
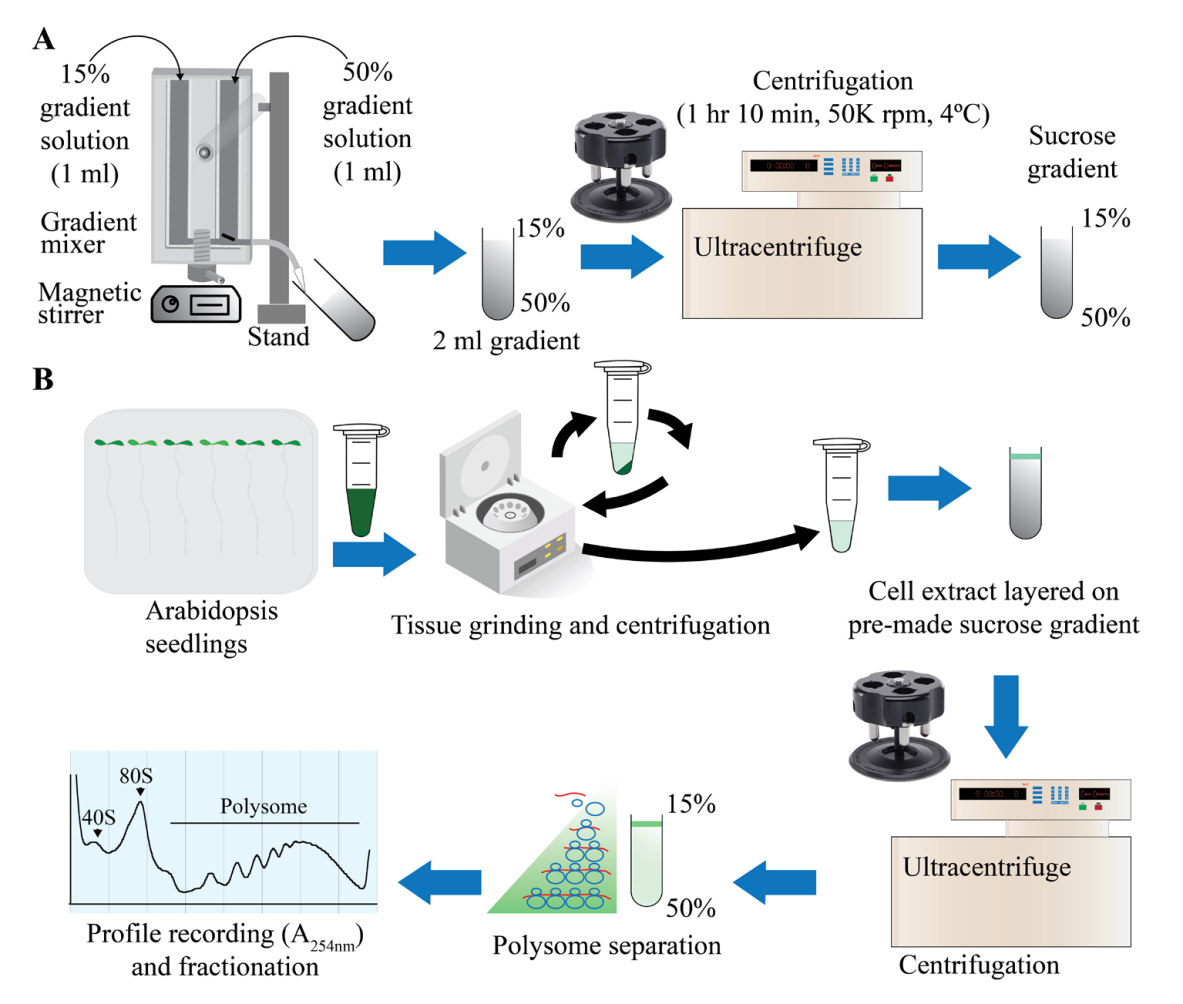
Graphical overview of polysome profiling using mini sucrose gradient. **A.** One milliliter each of 15% (w/v) and 50% (w/v) sucrose gradient solution is added to the individual chambers of the gradient maker. While mixing with a small magnetic stirrer in the 50% solution chamber, base station knob is turned to open position, allowing sucrose gradient solution to slowly flow through the outlet into a 2.2 mL gradient tube. After centrifugation at 50,000 rpm (213,626.2 *× g*) in a swinging bucket rotor for 70 min at 4 °C, the gradient tube is stored at 4 °C for the next steps. **B.** Cell extract from 12-day-old vertically grown *Arabidopsis thaliana* seedlings is centrifuged twice and 100 µL of supernatant is gently layered on the pre-made sucrose gradient from step A. After centrifugation as described in step A, polysome profile is obtained by feeding the gradient solution through an absorbance recorder (A254 nm). Eight (200 µL) fractions are collected for RNA and protein isolation.

## Background

Gene expression control is studied at multiple levels and, among these, translational control provides unparalleled insights about the functional readout of the genome in a spatiotemporal manner (Urquidi Camacho et al., 2020). For example, translatome studies provide highly valuable information that explain differences between the transcriptome (total mRNA pool) and the proteome (changes in total protein levels). Translation is governed by the dynamic distribution of cellular mRNAs into the non-translating and actively translating pool. The actively translating mRNAs loaded with multiple 80S ribosomes (polysome) are larger in size with higher density than the non-translating mRNAs, which are either free or associated with just the 40S ribosomal subunit. Polysome profiling by sucrose gradient centrifugation is a powerful technique for studying changes related to the distribution of mRNAs, as well as genome-wide effects on the translatome (Zuccotti and Modelska, 2016).

Traditionally, for translational control studies in plants (Mustroph et al., 2009; Layat et al., 2014; Missra et al., 2015; Yamasaki et al., 2015; Bai et al., 2017; Enganti et al., 2017; Lokdarshi et al., 2020a and 2020b), the method begins with the synthesis of a continuous sucrose gradient with variable density, for example, 15%–50% (w/v) sucrose gradient solution in a 5–10 mL centrifuge tube. Around 0.5–1 g of plant tissue material is ground and 0.5–1 mL of cellular lysate in polysome extraction buffer is gently layered onto the top of the gradient. The centrifuge tubes are spun at high speed for 3–4 h at 4 °C. RNA and protein components that are smaller in size and less dense (e.g., ribosome-*free* mRNAs, 40S ribosome subunit) travel less far into the gradient and therefore distribute at the top of the gradient, while larger species (e.g., 60S and 80S ribosome subunit, polysomes) travel farther into the gradient. After centrifugation, the gradient tube is assembled into a fractionator and 10–12 fractions (each with 1–1.2 mL) from the top (smaller, slower traveling) to bottom (bigger, faster traveling) are collected for RNA and protein extraction. Polysome profiles are constructed by measuring the optical density of the fractions while they are fed through an absorbance recorder (254 nm). Even though the traditional procedure continues to be a gold standard for studying translational control, this protocol requires ample starting material and suffers from being too lengthy, which further raises doubts over the quality of both RNA and the protein components. To overcome some of these drawbacks, we designed and successfully showed the use of a mini sucrose gradient for polysome profiling with *Arabidopsis thaliana* seedlings that has tremendous advantages, such as greatly reduced gradient synthesis and centrifuge times and low sample tissue requirement (Lokdarshi et al., 2020b and 2020c). The protocol described here can be easily adapted to a wide variety of organisms (e.g., bacteria, yeast, fungus, and plant and animal tissue) and even for the polysome profiling of organelles such as chloroplasts and mitochondria.

## Materials and Reagents

**Biological materials**

1. Twelve-day-old *Arabidopsis thaliana* ecotype Columbia (Col_0) seedlings

**Solutions**

1. DEPC-treated water
  Milli-Q water treated with 0.2% (v/v) DEPC for 24 h at room temperature and autoclaved for 20 min in a glass bottle. Cool down the bottled water at room temperature for 24 h before use. *(Can be stored indefinitely if the bottle is unopened)*
2. All reagent stocks (except sucrose solution, cycloheximide, RNase inhibitor and DTT) should be made in a glass beaker and stored in a 100 mL glass bottle in the dark at room temperature. *(Replace with new stock after three months)*
3. Cycloheximide (CHX, 50 mg/mL) stock is made by resuspending 50 mg of cycloheximide in 1 mL of ethanol in a 1.5 mL tube. Store stock solution at -20 °C. *(Replace with new stock after three months)*
4. Chloramphenicol (CHL, 50 mg/mL) stock is made by resuspending 50 mg of chloramphenicol in 1 mL of ethanol in a 1.5 mL tube. Store stock solution at -20 °C. *(Replace with new stock after three months)*
5. Dithiothreitol (DTT, 1 M) stock is made by resuspending 0.154 g of DTT in 1 mL of water in a 1.5 mL tube. Store stock solution at -20 °C. *(Replace with new stock after two months)*
6. Sucrose solutions made in a glass beaker need to be filtered through a 0.2 µm PVDF filter bottle and stored in the dark at 4 °C. *(Replace with new stock after one month. Before each use, always shake the bottle to check for any cloudy appearance. Discard and make new solutions as necessary)*
7. Punch solution should be made in a glass bottle and autoclaved for 20 min on liquid cycle. *(Can be stored indefinitely at room temperature in the dark)*
8. 100 mL of 1 M Tris-HCl (pH 8.4) is made by dissolving 12.14 g of Tris in 80 mL of deionized water and adjusting the pH to 8.4 using 0.1 N HCl. After attaining the desired pH, the volume of 1 M Tris-HCl solution is adjusted to final 100 mL using deionized water. *(Solution is stored at room temperature and it is recommended to replace with new stock after three months)*
9. Lysis buffer (1,000 µL in a 2 mL tube) (see Recipes)
10. 15% (or 50%) sucrose solution (100 mL) (see Recipes)
11. Punch solution (100 mL) (see Recipes)

## Recipes

1. **Lysis buffer (1,000 µL in a 2 mL tube)**
  Make fresh from stock solutions for each experiment.
  **Table:** 

Reagent	Final concentration	Amount
Tris-HCl (1 M, pH 8.4) KCl (0.5 M) MgCl_2_ (0.5 M) Deoxycholic acid (10%) Polyoxyethylene 10 tridecyl ether [20% (w/v)] Cycloheximide (50 mg/mL) RNase inhibitor (40 U/µL)	200 mM 50 mM 25 mM 1% (v/v) 2% (v/v) 50 µg/mL 40 U/mL	200 µL 50 µL 25 µL 100 µL 100 µL 1 µL 1 µL
H_2_O	n/a	523 µL
Total	n/a	1,000 µL
2. **15% (or 50%) sucrose solution (100 mL)**
  **Table:** 

Reagent	Final concentration	Amount
Tris-HCl (1 M, pH 8.4) KCl (0.5 M) MgCl_2_ (0.5 M) Sucrose	200 mM 50 mM 25 mM 15% or 50% (w/v)	20 mL 10 mL 5 mL 15 or 50 g
H_2_O	n/a	40 mL
Total	n/a	Make up final volume to 100 mL
3. **Punch solution (100 mL)**
*Note: Water should be added in increments of 10 mL. Allow the solution to mix completely before adding any more water. Sucrose will take a substantial volume when fully dissolved.
  **Table:** 

Reagent	Final concentration	Amount
Sucrose Bromophenol blue (0.1% (w/v)) Tris-HCl (1 M, pH 8.4)	60% (w/v) 0.02% (v/v) 1 mM	60 g 20 mL 100 µL
H_2_O	n/a	see note*
Total	n/a	100 mL

**Laboratory Supplies**

1. 1.5 and 2 mL tubes (Thermo Fisher, catalog numbers: AM12450 and AM12475)
2. Pipettes (200–1,000, 20–200, 2–20, and 0.2–2 µL)
3. DNase/RNase free tips (1,000, 200, 10 µL)
4. 1.5 mL tube rack
5. 7/16 × 13/8 in. (11 × 34 mm) 2.2 mL polypropylene tube (Beckman Coulter, catalog number: 347357)
6. 19 Gauge needle for gradient tube piercing (Fisher Scientific, catalog number: 14-826-52)
7. Parafilm (Fisher Scientific, catalog number: S37440)
8. Disposable bottle top filters (Thermo Fisher, catalog number: 597-4520)
9. Disposable bottle filter units (Thermo Fisher, catalog number: 568-0020)
10. Permanent ink markers
11. Clear tubing (6 inch in length) for gradient mixer outlet (1/16 in diameter)
12. Wipes (Fisher Scientific, catalog number: 06-666C)
13. Flat-tip forceps (Fisher Scientific, catalog number: 16-100-116)
14. Porcelain pestle and mortar (Fisher Scientific, catalog number: S27075)
15. Ice bucket (Styrofoam box can be used as an alternative)
16. Basic binder paper clip (medium)
17. Gloves
18. Stainless steel spatula with tapered end
19. Tris (Molecular Grade) (Promega, Fisher Scientific catalog number: PR-H5133)
20. KCl (Fisher Scientific, catalog number: AC418205000)
21. MgCl_2_ (Fisher Scientific, catalog number: M33-500)
22. Deoxycholic acid (Fisher Scientific, catalog number: BP349-100)
23. Polyoxyethylene 10 tridecyl ether (Millipore Sigma, catalog number: P2393-100G)
24. Cycloheximide (Millipore Sigma, catalog number:01810-G)
25. Chloramphenicol (Millipore Sigma, catalog number: 220551-25GM)
26. 40 U/mL RNase inhibitor (Promega, catalog number: N2515)
27. Sucrose, molecular biology grade (Millipore Sigma, catalog number: 573113)
28. Bromophenol blue (Fisher Scientific, catalog number: AAA1846909)
29. Dithiothreitol (DTT) (Thermo Fisher Scientific, catalog number: R0861)
30. Murashige and Skoog (MS) plant basal salt (MP Biomedicals, Fisher Scientific catalog number: ICN2623022)
31. RNaseZap^TM^ RNase decontamination solution (ThermoFisher, catalog number: AM9780)
32. Liquid nitrogen
33. Aluminum foil

## Equipment

1. Gradient mixer (Buchler Instruments). Catalog number for this specific product is not available as no vendor supports this product anymore. For custom manufacturing of the gradient mixer, see Figure 2.
2. Tabletop ultracentrifuge (Beckman Coulter, catalog number: A95761 or equivalent)
3. TLS-55 swinging-bucket rotor with four buckets for up to four gradients (Beckman Coulter, catalog number: 346936)
4. Gradient station or any equivalent gradient fractionators (Biocomp, catalog number: 153-002)
5. ISCO UA 5 100 absorbance/fluorescence monitor. Catalog number for this specific product is not available as the vendor is out of market. Alternative product information: BR-188 Density Gradient Fractionation System with Peak Chart Data Acquisition System (Brandel)
6. Tabletop centrifuge with rotor to hold 1.5 mL tube. If refrigerated centrifuge is not available, then a non-refrigerated centrifuge can be placed in a 4 °C cold room as an alternative
7. Magnetic plate stirrer
8. Magnetic stirrer bar (Fisher Scientific, catalog number: 14-513-93). Cut the magnetic stirrer bar into half with the plier and store the halves in a 1.5 mL tube (micro-stirrer bar).
*Note: Cleaning of this micro stirrer bar should be done with warm water and stored carefully.*
9. Weighing balance with 0.001 g accuracy
10. Vortex
11. Burette stand

**Software and Datasets**

The BR-188 Density Gradient Fractionation System comes with the Peak Chart Data Acquisition System Software. See note in section E to develop polysome profile without a Peak Chart Data Acquisition System Software.

## Procedure

- **Growing *Arabidopsis* seedling and tissue storage**
  This step is a standard procedure in plant biology and various recourses are available that outline the method for germinating *Arabidopsis* seeds on 1/2× MS plant media. After 12 days of growth, seedlings are harvested into a 1.5 mL tube, flash frozen in liquid nitrogen, and then stored at -80 °C.
*Note: It is recommended to use this sample within 1–2 months for best reproducibility of the experiment.*
- **Readiness of gradient maker and general setup (see Figure 1A and Figure 2A for details)**
*Note: Application of RNaseZap^TM^ RNase decontamination solution on gloves is recommended before beginning the following procedure.*
  1. One day prior to the polysome profiling experiment, rinse the gradient maker with diluted dish washing soap in warm tap water for 1–2 min and then wash with warm tap water for few minutes. Micro-stirrer bar needs to be cleaned with just warm water and dried with wipes.
  2. Rinse the gradient maker with DEPC-treated water twice and air dry on a few layers of wipes.
  3. Arrange the following items on a stable bench: pipettes, tips, burette stand, magnetic stirrer plate, magnetic stirrer bar, 2.2 mL polypropylene centrifuge tubes, 1.5 mL tube stand, trash cans for liquid and solid waste, and stock solutions of 15% and 50% sucrose, CHX, CHL, and DTT.
  4. Attach the gradient maker support rod to the burette stand holder and adjust the height of the holder such that the base of the gradient maker is ~0.5–1 inch above the magnetic stirrer plate.
  5. Add the micro-magnetic stirrer bar into the chamber connected to the outlet tubing (Well-II) and turn the magnetic stirrer plate ON to confirm rotation of the micro-stirrer bar.
  6. Turn the push-pull valve to face towards self, closing the gradient conduit between the mixing chambers (Well-II and Well-I).
  7. Clamp a binder clip to the outlet tubing (1 inch away from the gradient maker outlet valve).
  8. Attach a 10 µL pipette tip at the end of the outlet tubing using the broad side for the outlet tubing and the tip side for elution.
  9. CHL-CHX-DTT mix: Add 15 µL each of CHX and CHL stock and 1.5 µL of DTT stock into the same 1.5 mL tube and mix by vortexing at room temperature for 10–20 s. Store on ice.
    **Figure 2.**
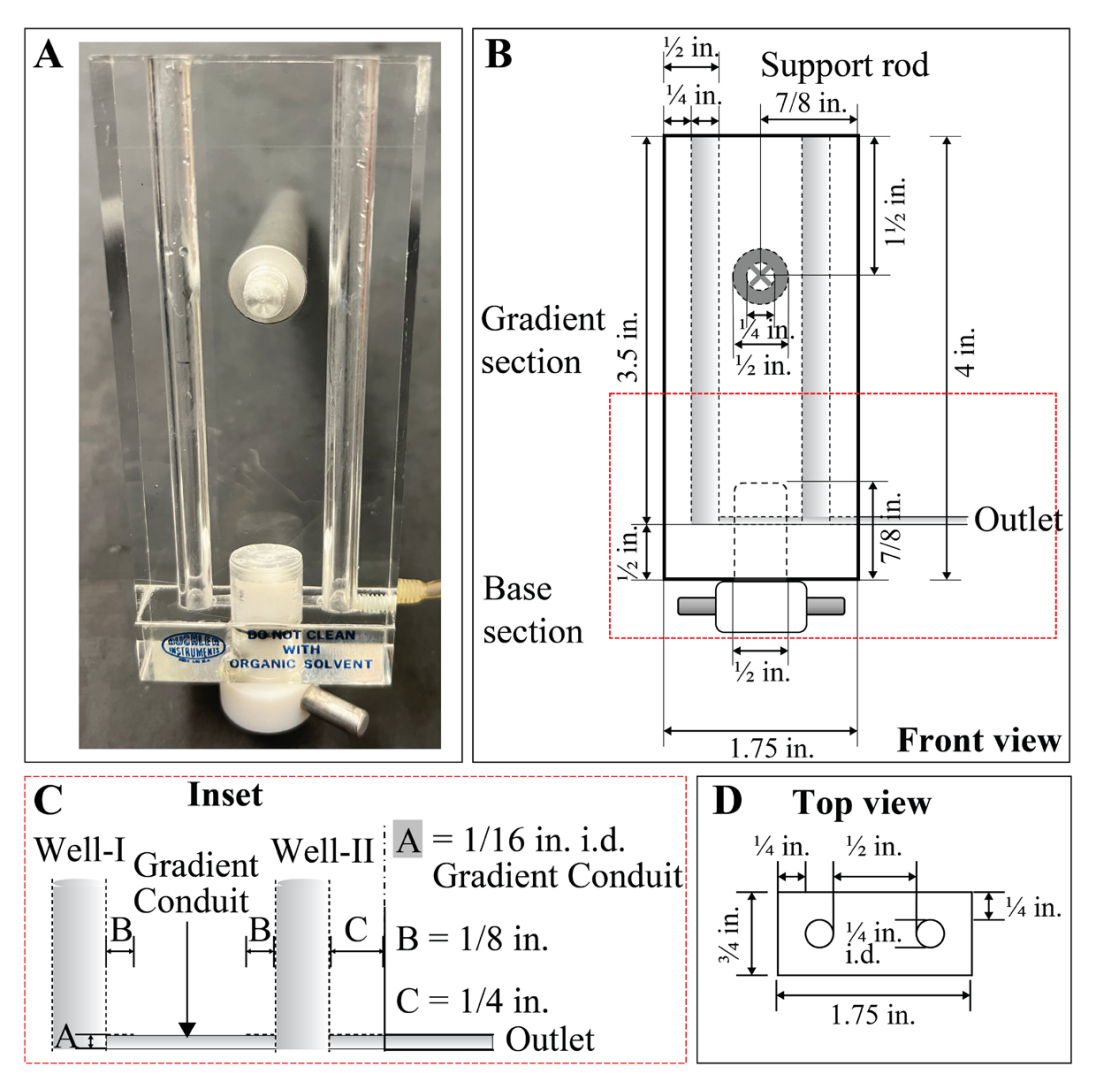
Schematics of the gradient mixer. **A.** Original view of the gradient mixer. The push-pull valve at the bottom of the apparatus is facing towards self, indicating closed connection between the two gradient chambers. **B–D.** Outline of the gradient mixer in different view angles showing specific measurements. Abbreviations: in = inches, i.d. = inner diameter
- **Preparation of 2 mL sucrose gradients for pre-run before polysome profiling**
  1. Add 2.1 µL of CHL-CHX-DTT mix along the side wall of each Well-I and II.
  2. Add 1 mL of 15% sucrose solution into Well-I and 1 mL of 50% sucrose solution in Well-II (magnetic stirrer should be rotating in Well-II while 50% sucrose solution is added to Well-II) (**see Figure 1A**).
  3. Remove the binder clamp gently to allow the 50% sucrose solution to flow temporarily approximately 1 cm into the outlet tubing. Quickly clamp back the tubing.
*Note: For a precise control over the flow of the 50% sucrose solution, the tip of the outlet tubing can be carefully inserted into the tip of a 200 µL tip attached to a 20–200 µL pipette. Once the clamp is removed, the pipette is adjusted to allow 50% sucrose solution exactly 1 cm into the outlet tubing. On completion of this step, clamp the tubing again and remove the 200 µL tip, and proceed to the next step.*
  4. Turn the push-pull valve to face horizontal position, opening the gradient conduit between the mixing chambers (Well-I and Well-II).
  5. Air bubbles trapped in the passage between the two wells need to be removed by sealing the top of the Well-I with a gloved finger and then slightly pressing down to increase the pressure.
*Note: This will release air bubbles trapped in the valve passage into the Well-II.*
  6. While making sure that the outlet is at the same height with the bottom of Well-II, place the tip of the outlet tubing into the bottom of a 2.2 mL ultracentrifuge tube and gently open the clamp. Lower the centrifuge tube slowly and let the solution flow along the walls of the centrifuge tube by gravity. Make sure that the solutions drain evenly from both Well-I and Well-II. When the solution in Well-II is almost over (~200 µL remaining), tilt the gradient maker clockwise until the base of the gradient maker touches the magnetic stirrer plate, while making sure the tip at the end of the outlet tube touches the top of the finished gradient.
*Note: This step will ensure all liquid flows out.*
  7. Place the finished gradient in a 1.5 mL tube rack and cover it with aluminum foil. Repeat the steps B6–9 and C1–6 for making more gradients.
  8. When all the gradients are done, adjust the weight of each gradient tube to 2.800 g by gently adding 15% sucrose solution on top of the finished gradient.
  9. For pre-run, place the gradient tube using forceps into a TLS-55 rotor bucket (pre-chilled at 4 °C) and make sure to match the weight with other tubes using 15% sucrose solution.
    *Note: It is important to spin all the four buckets of the rotor with equal weights*.
  10. Spin at 50,000 rpm (213,626.2 *× g*) for 70 min at 4 °C *(deceleration = 0, meaning no brakes)*.
  11. After the run, take the gradient tubes out using forceps and place in a 1.5 mL tube rack. Cover all the gradients with aluminum foil and store at 4 °C on a stable base without any shaking.
*Note: Pre-run gradients are good for up to 24 h.*
- **Cell lysis and sucrose density gradient centrifugation**
*Note: Two days prior to polysome profiling, wash mortars and pestles with dish soap and rinse with tap water. Use a new set for each sample to prevent cross contamination. Air dry and wrap one mortar with pestle as a pair with aluminum foil and autoclave for 30 min using dry setting. One day before the experiment (day of steps A and B), place the mortar-pestle set at -20 °C (or preferably at -80 °C). (This practice is done to reduce time spent for chilling the mortar-pestle set and save on liquid nitrogen.)*
*The term pre-chilled in the following section refers to the use of liquid nitrogen to achieve the coldest temperature.
  1. Add liquid nitrogen to the cold mortar-pestle until no boiling of liquid nitrogen is observed. Collect your tissue samples from the -80 °C freezer and keep them floating in a dewar with liquid nitrogen until it is their turn.
  2. Add the frozen tissue from the storage tube into the mortar and grind in liquid nitrogen to a fine powder using the pestle. *(This step is crucial and usually takes 4–5 min. After every 1 min, add some liquid nitrogen gently into the mortar to keep the ground material cold.)* It also helps to have the mortar sit inside a Styrofoam box that is kept cold with liquid nitrogen.
  3. Using a pre-chilled spatula, weigh 150 mg of pulverized tissue powder in a pre-chilled 1.5 mL tube.
*Note: During waiting periods, 1.5 mL tube with open cap should be kept in a rack that is placed inside a Styrofoam container with some liquid nitrogen. The cap is left open to allow liquid nitrogen to evaporate. Cover the container to avoid condensation buildup on the top of the 1.5 mL tube.*
  4. Add 100 µL of freshly prepared lysis buffer (see Recipes) into the 1.5 mL tube with tissue powder and place the tube in a new rack kept at room temperature.
  5. After 40–50 s, close the cap of the tube and vortex at highest setting for 1 min at room temperature. Repeat this step twice to achieve complete homogeneity.
  6. Spin the tube at 21, 000 *× g* for 5 min at 4 °C.
  7. Transfer 125 µL of the supernatant into a new 1.5 mL tube and repeat centrifugation at 21,000 *× g* for 5 min at 4 °C.
  8. Carefully layer 100 µL of the supernatant from step 7 onto the pre-made 15–50% sucrose gradient (see section C).
*Notes:*
    - *Tubes can be numbered with a marker at this stage.*
    - *For a blank gradient, layer 100 µL of lysis buffer on a pre-made 15%–50% sucrose gradient.*
  9. Place the gradient tube using forceps into a TLS-55 rotor bucket (chilled at 4 °C) and make sure to match the weight with other tubes. 15% sucrose solution can be used for this purpose.
    *Note: It is very important to use all the four rotor buckets with equal weights*.
  10. Spin at 50,000 rpm (213,626.2 *× g*) for 70 min at 4 °C *(deceleration = 0, meaning no brakes).*
  11. Take the gradient tubes out using forceps and place the tubes in a 1.5 mL tube rack (Figure 3A). Cover all the gradients with aluminum foil and place on ice.
- **Polysome profiling and gradient fractionation**
*Note: To begin Section E, the gradient should be clamped down in the gradient fractionator and sealed against the top assembly with the outlet tube. (Refer to manufacturer’s manual for details.) The general idea is that the gradient tube gets pierced at the bottom and the heavy push solution is pumped into the bottom of the gradient tube. This way, the gradient is displaced up through the outlet tube at the top into the absorbance recorder and finally out for fractionation.*
*After each gradient run, the fractionator tubing should be washed with 50 mL of warm water by siphoning it from one end. Dry both the inlet and outlet with wipes and apply vacuum to get rid of any residual water in the fractionator tubing. The baseline absorbance (A_254nm_) should return to zero.*
  1. Run the punch solution through the fractionator tubing at lowest speed (for example, 375 μL/min in the ISCO Teledyne gradient fractionator). Continue pumping the punch solution until it comes out of the piercing needle.
  2. Carefully wrap a small piece of parafilm (2 cm wide) around the circumference of a 2.2 mL tube with just water and load the tube into the holder of the gradient fractionator.
*Note: Parafilm is installed for better grip of the tube with the adapter for gradient fractionator.*
  3. Rotate the collector piston to pierce the gradient tube and start the pump as described in step E1 (Figure 3B).
  4. As the water starts to come out of the fractionator tubing, adjust the absorbance (A_254nm_) to zero for recording baseline at sensitivity of 0.5 in the ISCO Teledyne gradient fractionator.
  5. On completion of the water tube run, take the tube out (see step E6) and load the blank gradient tube (see step D8b) for recording blank reading (Figure 3C).
  6. After each gradient run, the fractionator tubing should be washed with 50 mL of warm water by siphoning it from one end. Dry both the inlet and outlet with wipes and apply vacuum to get rid of any residual water in the fractionator tubing. The baseline absorbance (A_254nm_) should return to zero.
  7. After blank gradient recording is complete, take the tube out and load the first sample gradient tube (Figure 3A). Collect eight 200 µL fractions in individual 1.5 mL tubes. Place tubes on ice immediately after each fraction collection.
    **Figure 3.**
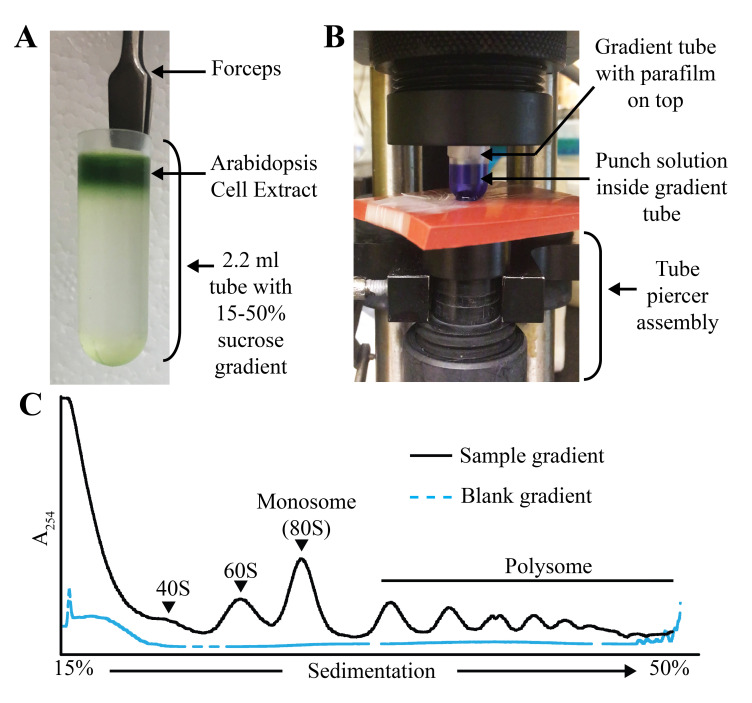
Miniature gradient tube setup and polysome profile. A. Gradient tube with sample (*Arabidopsis* cell extract) after ultracentrifugation. B. Setup of the gradient tube in the ISCO Teledyne gradient fractionator with punch solution inside the tube. C. Example of 15%–50% miniature sucrose gradient. The position of the 40S, 60S, monosome (80S), and the polysome is indicated on the sample gradient profile.

## Notes

If a gradient fractionator is not available, individual fractions can be collected by carefully aliquoting 200 µL of the finished gradient from the top using a 20–200 µL pipette. For polysome profile, RNA extracted from these fractions can be quantified using a spectrophotometer and plotted with x-axis as fraction # and y-axis representing absorbance.
